# Investigation of conduction mechanisms and permittivity–conductivity correlation in a Gd-based perovskite structure

**DOI:** 10.1039/d3ra08703d

**Published:** 2024-01-30

**Authors:** Khouloud Moualhi, Youssef Moualhi, Mouldi Zouaoui

**Affiliations:** a Faculty of Science of Bizerte, Laboratory of Physics of Materials: Structure and Property LR01ES15, University of Carthage Tunisia zouaoui.mouldi@fsb.u-carthage.tn Khouloud.Moualhi@fsb.u-carthage.tn

## Abstract

Currently, the development of perovskites has required a lot of attention for fundamental investigation and electronic devices. The present study reported the electrical conductivity and the dielectric properties of a GdCa_2_Cu_3_O_*δ*_ (GdCaCuO) system prepared *via* the solid-state reaction method. The X-ray diffraction results indicate that GdCaCuO crystallizes in the tetragonal perovskite structure. The studied compound reveals elevated dielectric permittivity and significant electrical phenomena including the influence of multiple conduction and relaxation mechanisms on the transport of charges. From the impedance results, it is observed that the electrical and dielectric properties of the studied compound are governed by the contribution of the grain and the grain boundary regions. The Nyquist results and the blocking factor (*α*_R_) were used to confirm the aforementioned properties. From the modulus results, we confirm again the presence of multiple relaxation processes and various types of polarization effects. The semiconductor character of the GdCaCuO ceramic is due to the activation of hopping conduction processes within the DC regime. Consequently, both the variable range hopping and the small polaron hopping models are used at low- and high-temperature ranges, respectively. At various temperatures, the conductivity spectra of the GdCaCuO ceramic conform to the double Jonscher power law. The power law behavior is attributed to the activation of hopping and tunneling conduction mechanisms. The presence of large dielectric dispersion in GdCaCuO is observed at low temperatures and decreases rapidly against increasing temperatures.

## Abstract 2

Currently, the development of perovskites has required a lot of attention for fundamental investigation and electronic devices

## Graphical abstract

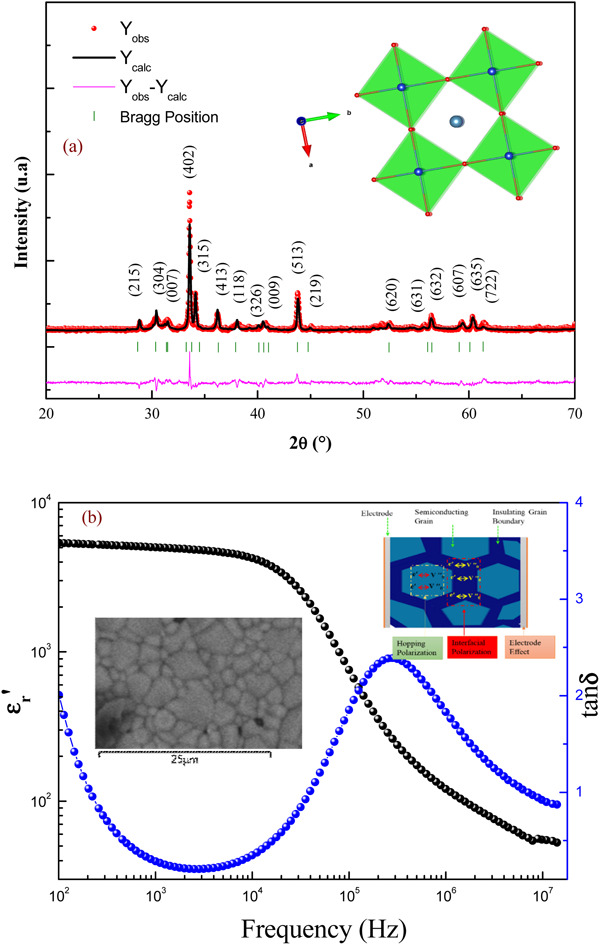

## 1 Introduction

Nowadays, scientists have turned their attention to developing the efficiency of perovskites to replace fossil fuel energy funds with cleaner renewable sources.^1–4^ Thus, research groups have sought to develop new compounds with elevated dielectric permittivity and critical electrical properties.^5–7^ Numerous kinds of research focused on the origin of the transport properties and the impact of the materials microstructure on some critical dielectric properties.^1,5^ Among functional materials, CaCu_3_Ti_4_O_12_ perovskite has long been attracting significant consideration given its various exceptional physical characteristics such as the elevated dielectric characteristics, the contribution of several conduction processes to the transport properties of this kind of material, and the irreversibility field.^5^ Both the electrical and dielectric properties of the ceramic compounds are sensitive to dipolar species and localized charges, which are the responsible parameters for determining the strength, the kinematics, and the charge carrier interactions.^4^ Therefore, it is challenging to correlate the structural, dielectric, and electrical properties of ceramics to produce multifunctional compounds and use them in future applications.^8^ Due to their high permittivity (*ε*′) and its motivating performance, low dielectric loss, and the observed excellent temperature stability of *ε*′ over a large temperature domain, GdCaCuO complex ceramics have been recently proposed to fabricate Y5R and Y6R ceramic capacitors.^8^ Similar ceramics like CaCu_3_Ti_4_O_12_ are used in the fields of energy-storage devices and microelectronics (sensors, pass capacitors, dynamic random access memory devices, actuators, and high-energy-storage devices).

In the literature, the electrical and dielectric responses of ceramic materials are attributed to the major role of polarization effects and the microstructure properties when the material is exposed to an external electrical field.^5^ Accordingly, the investigation of electrical and dielectric responses gives an idea about charge storage, charge density, and mobility.^2^ It permits the identification of the conduction mechanisms that govern the transport and the relaxation phenomena. Similarly, impedance spectroscopy characterization gives significant information about the frequency range in which each polarization contribution and the conduction mechanisms are activated.^9^ For several oxide materials, it is found that electrical conductivity and charge storage mechanisms depend on temperature, frequency, impurities, and crystal defects.^10^ According to Maxwell–Wagner's model and Koops' theory,^11^ conducting grain regions are active at a higher frequency range. The frequency-dependent electrical conductivity of oxide systems usually obeys the Jonscher power law,^12,13^ and the Bruce model.^14^ Other electrical condition laws like the super-linear and the nearly constant laws have been employed for simple perovskites.^15^

For the studied system, the electrical conductivity spectra and the dielectric properties at various temperatures (from 220 to 450 K) are investigated based on numerous polarization, relaxation, and conduction models. For instance, the conductivity spectra were analyzed using the universal Bruce law. Accordingly, the power law variation has been explained using numerous hopping and tunneling conduction mechanisms like the overlapping large polaron tunneling, the non-overlapping small polaron tunneling, the correlated barrier hopping, and the quantum mechanical tunneling mechanisms. In the DC regime, the semiconductor behavior that is noticeable in numerous classes of materials has been usually investigated based on Mott's theory.^16–18^ Accordingly, both the small polaron hopping and the variable range hopping conduction mechanisms have been proposed to examine the origin of some transport properties. The presence of numerous relaxation phenomena and the important contribution of numerous types of polarization effects have been investigated using both impedance and modulus analysis. The dielectric properties of GdCaCuO are discussed using the well-known intrinsic IBLC polarization, a hopping mechanism, oxygen vacancy ionization, and metal vacancy complexes that co-exist and contribute to the appearance of elevated permittivity and encouraged conductivity in GdCaCuO.

## 2 Experimental section

The compound with structure GdCaCuO was synthesized, at high temperatures, using a solid-state reaction method. A detailed explanation of the elaboration steps and the optimization of the preparation conditions that give the best electrical and dielectric performances of the material are mentioned in our previous work.^8^ Accordingly, a stoichiometric mixture of fine powders of Gd_2_O_3_ (99.9%), CaCO_3_ (99.9%), and CuO (99.9%) was employed to prepare the sample. The oxides and carbonate precursor were intimately blended in an agate mortar. The mixed powders were pressed into pellets and calcined at 950 °C, which gives the best dielectric properties (elevated dielectric constant, and lower loss tangent) in the GdCaCuO. The synthesis takes place in two sintering cycles separated from each other by milling and pressing into pellets.

Cristal structure (lattice and space group), homogeneity, and phase purity of the prepared sample were recently discussed in our previous work in which we found that the structural phase formation of the material is observed for the sintering temperature of 950 °C obtained using an X-ray diffraction (XRD) characterization. The structural lattices were deduced using the Rietveld refinements. To examine the electrical proprieties of the sample, the powder was pressed into a pellet of 1 cm diameter and approximately 0.5 mm thickness. On both sides of the pellet, a thin Ag film was deposited using a circular mask of 1 cm in diameter. An Agilent Hewlett–Packard HP 4192 analyzer has been used to measure the conductivity. All measurements were conducted at different temperatures in the frequency range 10 Hz to 13 MHz.

## 3 Results and discussions

### 3.1 Structural and dielectric proprieties in GdCaCuO

**Fig. 1:**
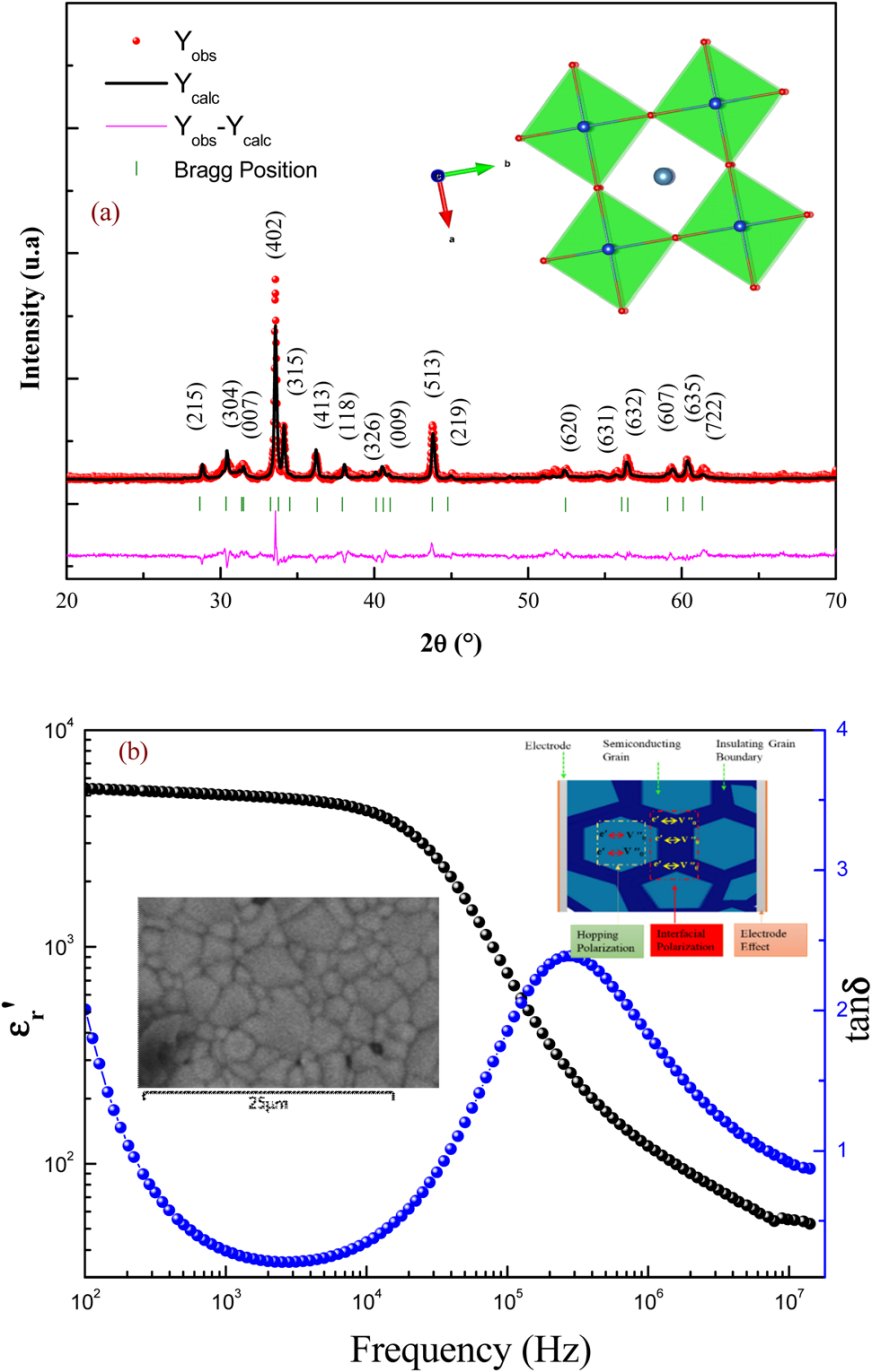
Rietveld refinement of the XRD pattern of GdCaCuO ceramic (a), the tetragonal unit cell structure of the sample (atomic positions are shown in parentheses). Frequency dependence of the dielectric constant and loss for GdCaCuO ceramics at room temperature (Insert: SEM micrograph of CCTO ceramic and the physical model of electronic motion) (b).

The crystallographic quality of the prepared compound was determined using X-ray powder diffraction. Fig. 1(a) displays the XRD pattern for the prepared sample with Rietveld refinement profiles attained using FullProf software. The determination of lattice parameters and the identification of the phase of the sample were carried out by the refinement in our previous work.^8^ The reported result indicates that the elaborated ceramic crystallizes in the tetragonal unit cell with a *P*4/*mmm* space group. Using the Rietveld refinement profiles, the obtained lattice parameters are *a* = *b*= 11.084(9) Å, and *c* = 19.834(4) Å.

Morphology of the sintered surfaces of the ceramics annealed at 950 °C was examined by scanning electron microscopy (SEM). The sample showed non-uniform grain sizes and the mean grain sizes of the sintered pellets were found to be 3 μm (inset Fig. 1(b)). The grain was well-crystallized with clear grain boundaries. The microstructure of the compound has a significant impact on its dielectric properties. The dielectric properties [the dielectric constant (*ε*′) and the dielectric loss (tan *δ*)] of GdCaCuO ceramics at room temperature are presented in Fig. 1(b). The sample has a high dielectric permittivity and stability over a wide frequency range [10^2^–10^4^ Hz]. At 3 kHz, the dielectric constant is about 5000 with a loss of 0.2. It has been observed that there is a relaxation process that occurs at 200 kHz. Due to this relaxation process, the dielectric constant of the ceramics decreases at high frequency. The dielectric behaviors of the investigated ceramic are similar to the reported results in the CaCu_3_Ti_4_O_12_ compound.^4,5^

The dielectric properties of the ceramics are strongly affected by the microstructure (size and uniformity of grain size) and the nature of grains and grain boundaries. The large dielectric response was reported to be caused by the electrically heterogeneous nature of semiconducting grains and highly insulating grain boundaries. The internal barrier layer capacitance (IBLC) model based on heterogeneous microstructure has been frequently employed to explain the giant dielectric permittivity response of GdCaCuO ceramics.^8^ Meanwhile, the formation of a space charge zone was a common phenomenon at grain boundaries. The relaxation processes of GdCaCuO ceramics were analyzed to reveal the dynamic charge carriers and the origin of the interfacial or Maxwell–Wagner (MW) polarizations (insert: Fig. 1(b)).

In the literature, the electrical and dielectric properties of the copper oxide systems are linked to the hopping of electrons in copper–oxygen vacancy complexes. In addition, the occurrence of elevated intrinsic dielectric characteristics is related to the strong interfacial polarization (IBLC) that results from the presence of insulating grain-boundary barrier layers. For copper dielectric ceramics, the grain boundary regions are composed of oxygen vacancy ionizations, copper oxidation, defects in the junction, and space charges that support their high dielectric permittivity. Using the Kroger and Vink notation, the oxygen ionization relation is given by the following expression:^19,20^1
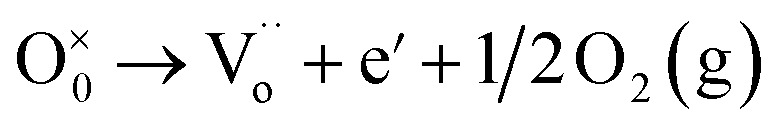

2 Cu^3+^ + 1e′ → Cu^2+^

where, 
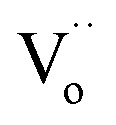
 is the two dots indicate doubly positively charged oxygen vacancy with respect to the neutral lattice.

For the studied material, oxygen vacancies are easily produced, as the breaking of Cu–O bonds does not require an elevated energy. Usually, oxygen vacancies could be otherwise charged 
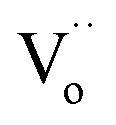
, 
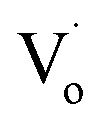
, V_o_, 
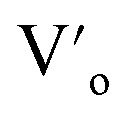
, 
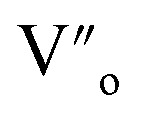
 (while two single quotation marks means negatively charged oxygen vacancy with respect to the neutral lattice and the two dots indicate doubly positively charged oxygen vacancy). In addition, the investigated GdCaCuO system reveals a positively charged oxygen vacancies that are compensated by free electrons or aliovalent metal ions. Therefore, one can reveal metal–oxygen vacancy complexes like 
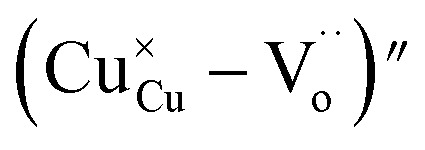
 and 
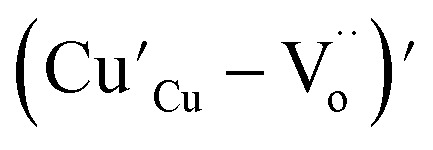
, the latter one in smaller number due to the oxidation aliovalency of the Cu element. This type of oxidation and the existence of the Cu^2+^/Cu^3+^ states are confirmed in our previous work using the X-ray photoelectron spectroscopy.^8^

It's interesting to note that the dielectric constant of the GdCaCuO ceramic at 0.5 kHz, 1 kHz, and 10 kHz is almost unaffected by the temperature studied in our previous study.^8^ To qualify these ceramics as important dielectric material components for capacitor fabrication of various types, such as Y5R and Y6R, this primary observation is of utmost importance.^8^

We are interested in the following work on the relationship between dielectric proprieties and conductivity in low and high temperatures. However, in semiconducting materials, localized charge carriers hopping between spatially fluctuating lattice potentials not only produce conductivity but also give rise to dipolar effects. In fact, the polarization, in particular, of the oxygen vacancy-related defects is accompanied by the release of carriers which results in the global conduction. The analyses and information provided by complex impedance spectroscopy are used to study the different conduction mechanisms as a function of frequency and temperature, and the explicit contribution of grains and grain boundaries to the overall electrical performance of GdCaCuO perovskite.

### 3.2 Impedance study

For the aim to investigate the contribution of the grains and grain boundaries as well as the electrodes in the electrical proprieties of the studied compound, an electrical equivalent circuit has modeled the electrical response (*Z*′′ *versus Z*′) of GdCaCuO. For each temperature value, the following relation describes the complex impedance function:

3 Z*(*ω*,*T*) = *Z*′(*ω*,*T*) − *jZ*′′(*ω*,*T*)

**Fig. 2:**
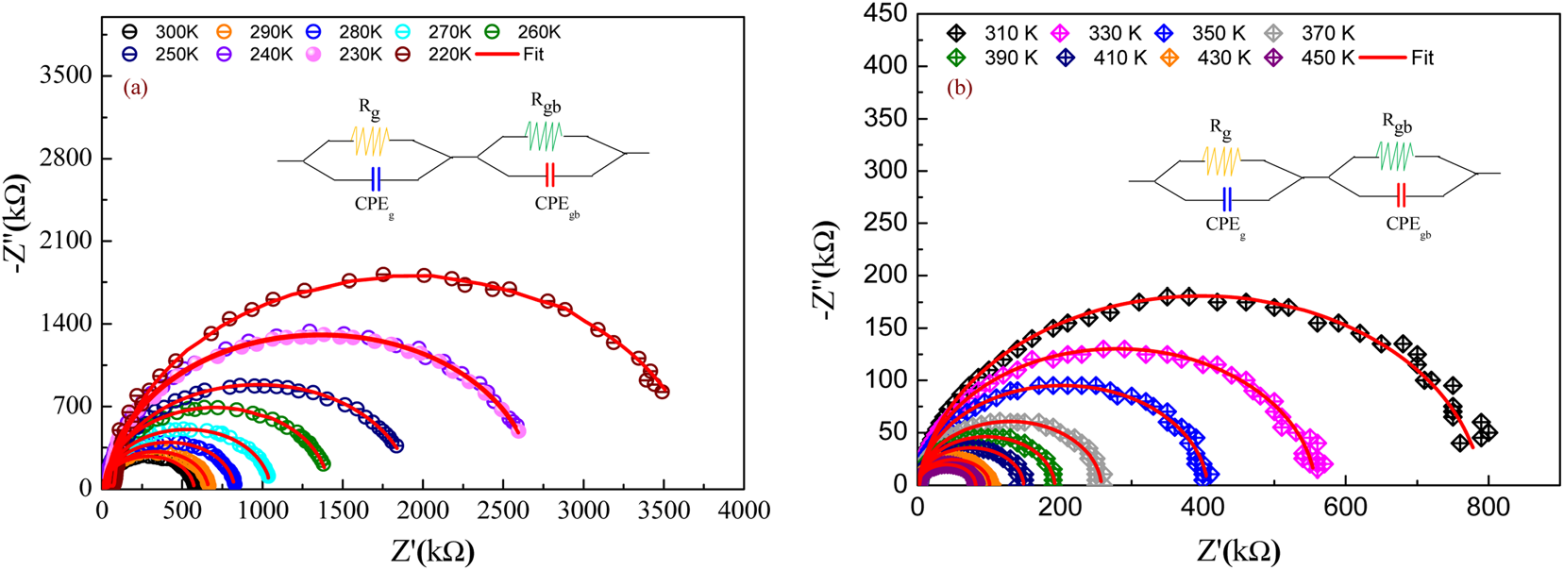
Nyquist plot of the prepared sample at different temperature ranges: from 220 K to 300 K (a) and, from 310 K to 450 K (b) (solid line represent theoretical fit). Inset illustrations of the equivalent circuit.

*Z*′ is the real part of the impedance termed resistance, and *Z*′′ is the imaginary part of the impedance termed reactance. To identify the contribution of grains, grain boundaries, and electrode effects, detected in electrical and dielectric properties, we determine from experimental data the real and imaginary parts of the impedance spectroscopy, and we plot *Z*′′ *versus Z*′. Over the explored temperature range (from *T* = 220 K to *T* = 450 K), the Nyquist plots (*Z*′′ *versus Z*′) of GdCaCuO are presented in Fig. 2(a and b). Complex impedance spectra are often espoused in analyzing the effects of the grain and the grain boundary (GBs) resistances. To show the important role of the contribution of grains and grain boundary in the electrical conductivity of the studied sample, we need to model the sample by an equivalent electrical circuit with the Z-View software. The most appropriate equivalent circuit, which fits the experimental data, is designed in the insets of Fig. 2 (where *R*_gb_ and *R*_g_ are the grain boundary and grain resistances, respectively, and CPE_gb_ and CPE_g_ are the constant phase elements of the grain boundary and grains, respectively).^21–23^ This model allowed us to connect the electrical properties and microstructure of the studied sample.

**Fig. 3:**
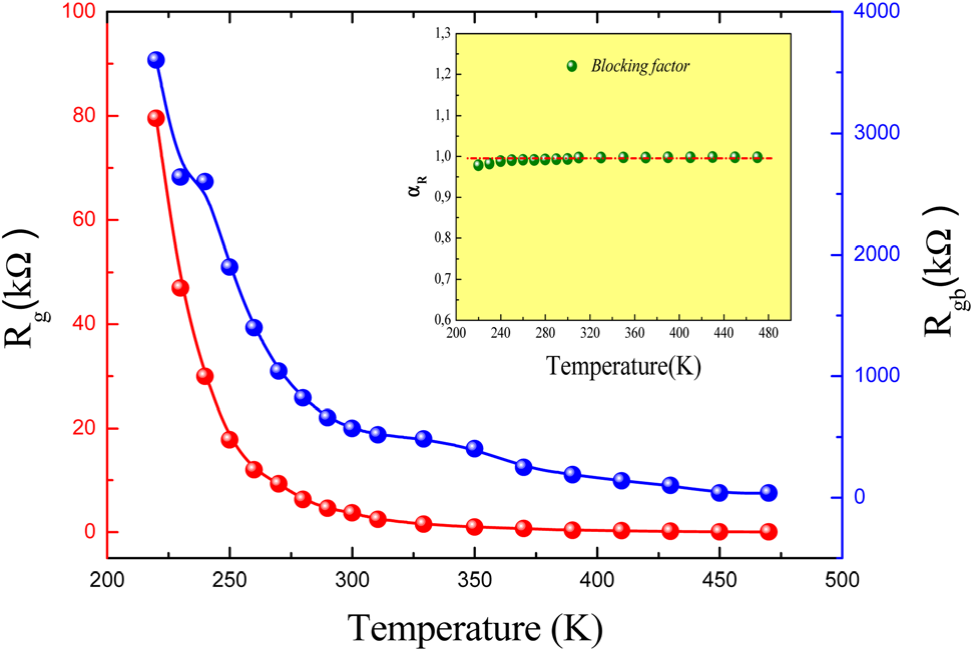
Resistance of grains (*R*_g_) and grain boundaries as a function of temperature deduced from the equivalent circuit (inset: variation of the grain boundary blocking factor as a function of temperature).

The thermal decrease of the electrical resistance supported the semiconductor nature of the synthesized ceramic. At high temperatures, it is observed that both *R*_g_ and *R*_gb_ decrease as temperature increases indicating that the electrical conduction in the prepared sample is thermally activated (Fig. 3). The influence of the grain boundary contribution to the total conductivity of the compound is estimated through the temperature dependence of the blocking factor *α*_R_ revealed in inset of Fig. 3, which is deduced using the following relation:^24^4
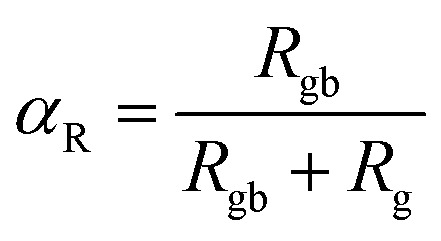


**Fig. 4:**
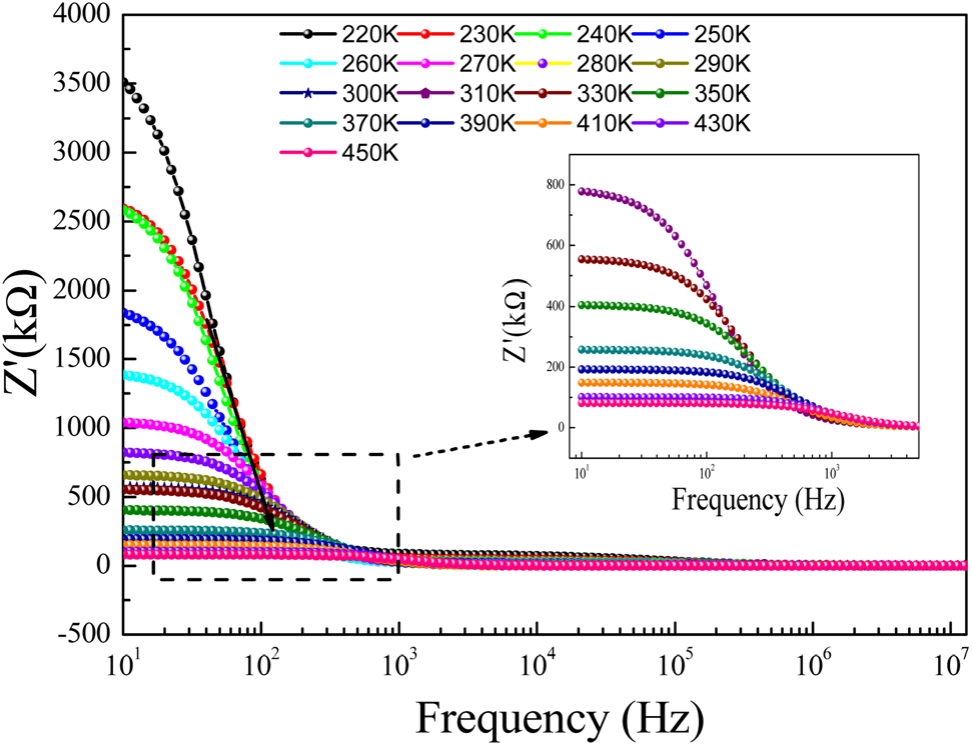
Real part (*Z*′) of impedance *versus* frequency for a temperature range, 220–300 K, and (inset) 310–450 K.

The *R*_g_ and *R*_gb_ resistances are obtained from the Nyquist plots, at various temperature values. In the literature, it is found that *α*_R_ varies between zero and one. For the studied compound, the grain boundary effects govern the electrical transport properties when *α*_R_ ∼ 1. In addition, when *α*_R_ tends to zero, the grain resistance controls the electrical characteristics of the ceramic compounds. The temperature dependence of the blocking factor for the studied compound indicates that *α*_R_ reveals values near to one, confirming that the grain boundary contributions govern the transport properties. The same results were observed in the oxide systems. From the reported results in Fig. 4, we found that the temperature increase is associated with a decrease in *Z*′. Beyond a certain frequency value, the significant *Z*′ decreases can be explained by the decrease in the density of trapped charges and the improvement of the mobility of load carriers. At the high-frequency region, *Z*′ merges and remains constant, confirming the existence of space charges in the studied sample.

**Fig. 5:**
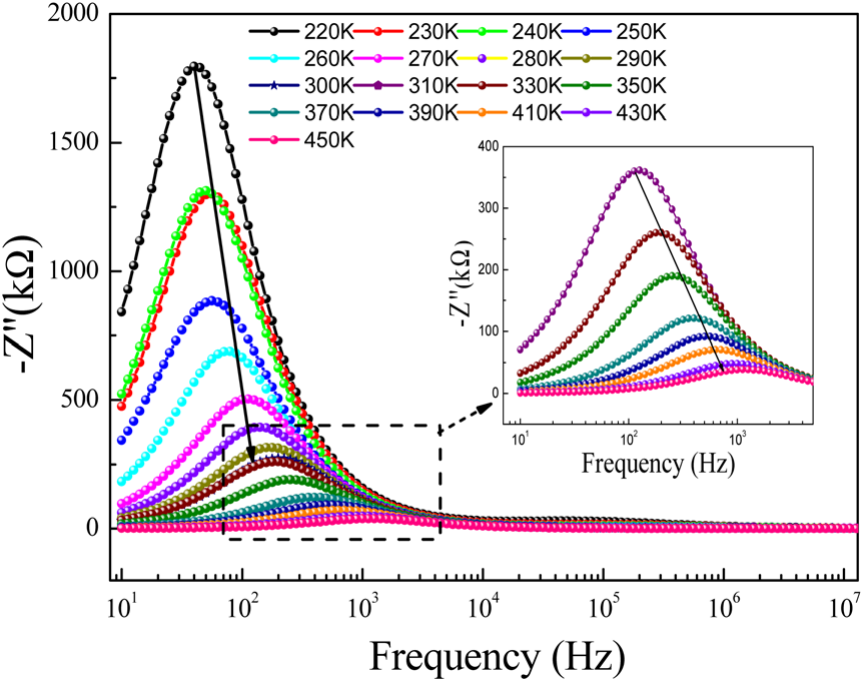
Imaginary part (−*Z*′′) of impedance *versus* frequency for a temperature range 220–300 K, and (inset) 310–450 K.

For the prepared compound, (GdCaCuO) the frequency dependence of the imaginary part of impedance (*Z*′′) at different temperatures is illustrated in Fig. 5. The spectra are defined by the presence of a relaxation peak at an exceptional frequency named the relaxation frequency (*f*_r_). By increasing the temperature, the relaxation peak is found to shift to higher frequencies, suggesting the existence of relaxation phenomena.^25^ For each temperature value, the relaxation frequency *f*_r_ value can be used to calculate the relaxation time *τ* using the relation: 
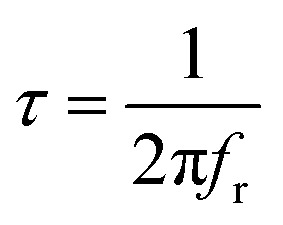
.

**Fig. 6:**
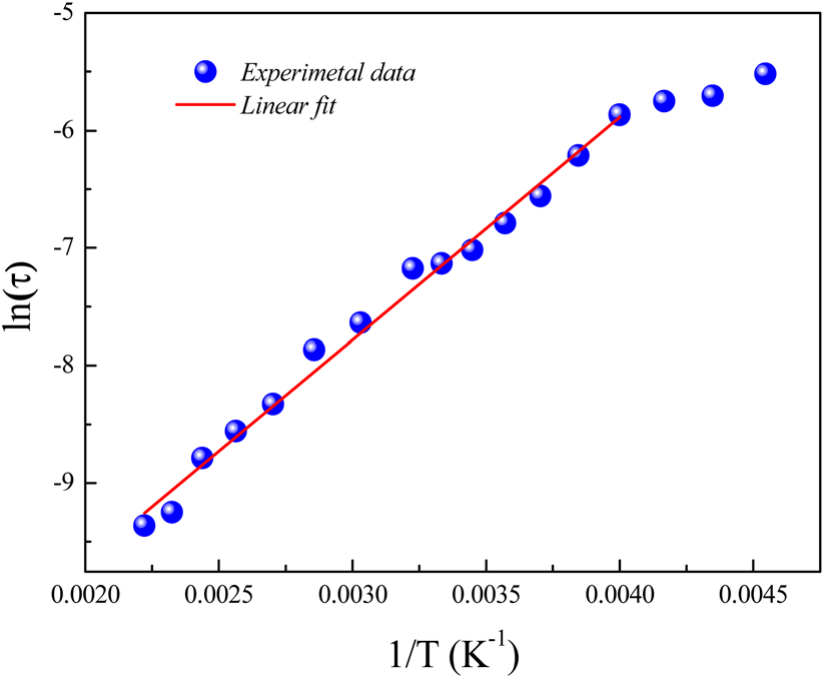
Evolution of ln(*τ*) as a function of the inverse of the temperature for the studied compound and the obtained activation energy value.

Fig. 6 displays the variation of ln(*τ*) *versus* the reciprocal temperature of the GdCaCuO system. In such case, the experimental results of the relaxation time were evaluated *via* Arrhenius's expression:5
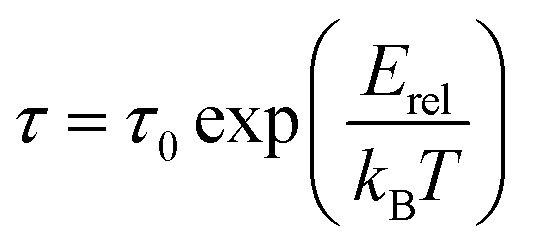


*E*_rel_ is the activation energy for the relaxation time and *τ*_0_ is the free exponential factor. The deduced value of the activation energy of the thermal dependence of the relaxation time is *E*_rel_ = 0.16 eV.

### 3.3 Modulus study

Electric modulus is suitable for investigating oxide materials' conduction and relaxation behaviors. Thus, the electrical modulus investigation can be employed to identify the presence of multiple relaxations, conduction processes, and charge transport mechanisms in the oxide materials. The frequency-dependent complex electrical modulus is expressed by the following relationship:

6 M*(*ω*) = *M*′(*ω*) + *jM*′′(*ω*) = *jωC*_0_Z*(*ω*)

*C*_0_ is the geometrical capacitance of the material. The complex impedance (Z*) rapports the grains and grain boundary resistances (*R*_g_ and *R*_gb_) and capacitances (*C*_g_ and *C*_gb_) that could be deduced from the equivalent electric circuit. *M*′ and *M*′′ are the real and the imaginary parts of the electrical modulus, respectively.

**Fig. 7:**
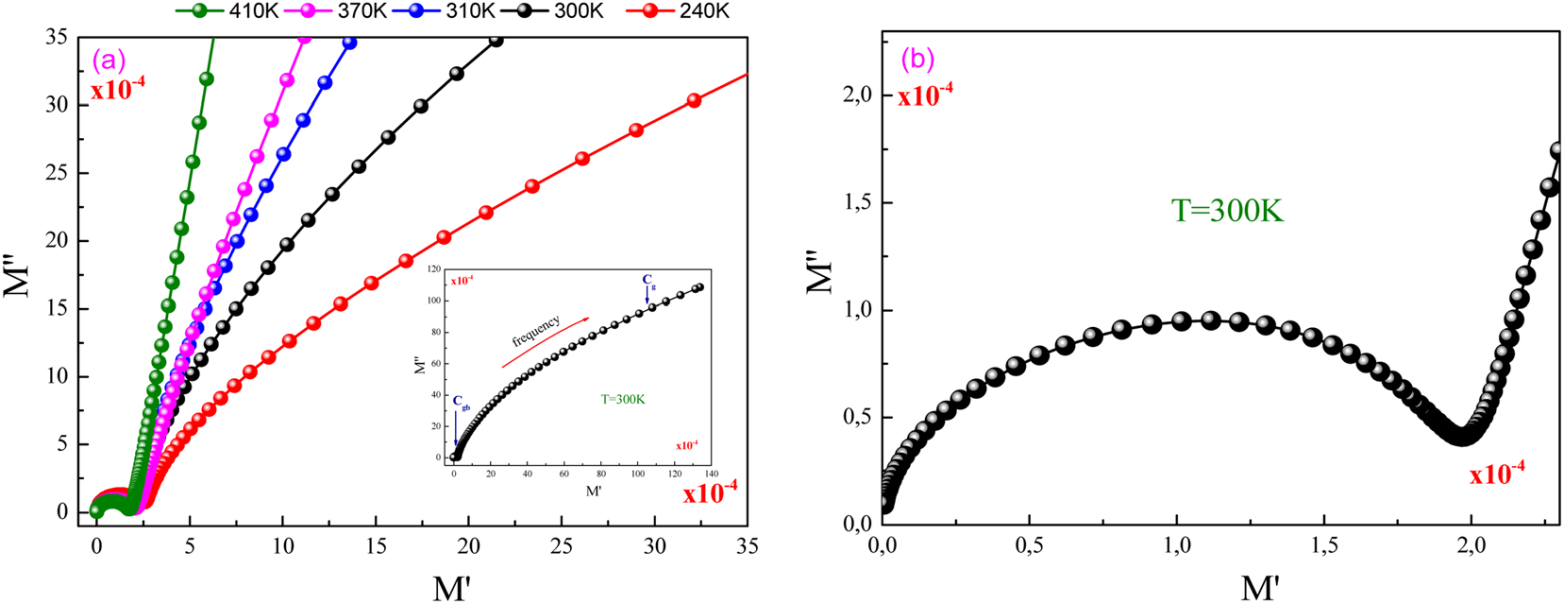
(a and b) Nyquist plots of modulus spectra between real (*M*′) and imaginary (*M*′′) parts for different temperatures.

Fig. 7(a) displays the evolution of *M*′′ *versus M*′ (Nyquist plots) at different temperature values for the investigated GdCaCuO compound. For each Nyquist plot, the right semicircle corresponds to the grain capacitance (*C*_g_) contribution, while the left one corresponds to the grain boundaries capacitance. For the studied compound, the non-Debye relaxation type is confirmed by the fact that the center of the semi-circular arc lies a little below the abscissa axis (Fig. 7(b)).^26^

**Fig. 8:**
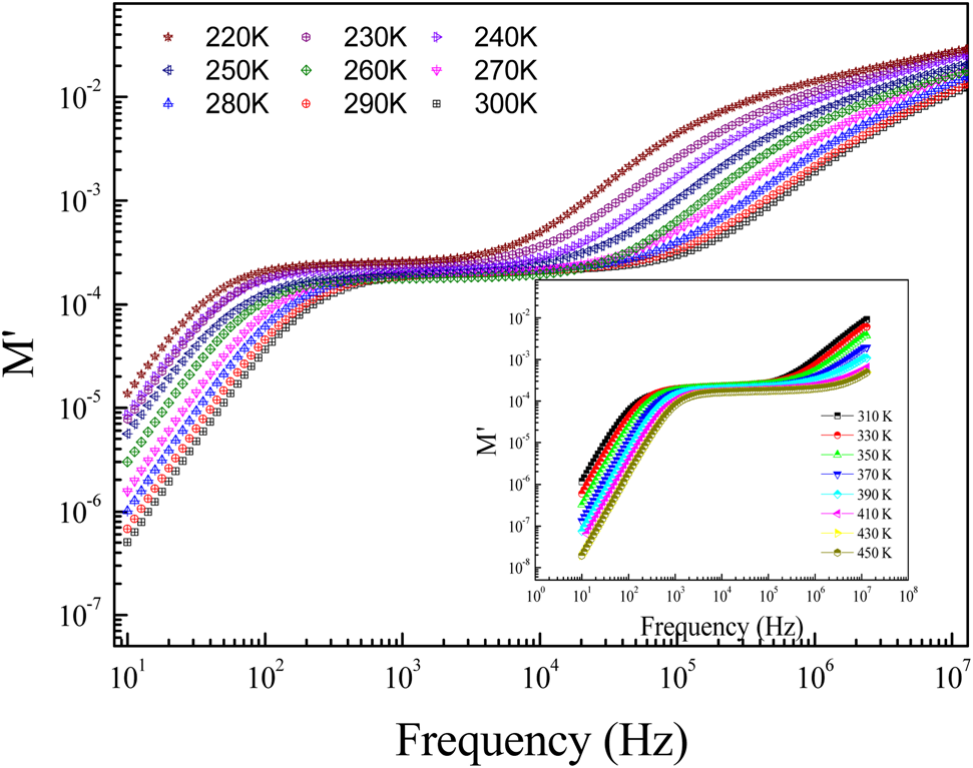
Spectra of the real part *M*′ of the electric modulus at selected temperature values.

Fig. 8 displays the evolution of the real parts of the electrical modulus (*M*′) as a function of the frequency at numerous temperature values. In the first temperature range, it is noticeable from the curve that the real parts of the electrical modulus (*M*′) increase with increasing frequency and practically take nearly a constant value beyond 1 kHz. The increases of *M*′ with increasing frequency, at low frequencies, can be explained by the significant electrode and/or ionic polarization.^27^ At high-range frequencies, we found that *M*′ reveals a dispersion variation that can arise due to the conductivity relaxation in the studied ceramic. In the intermediate frequency range, *M*′ displays a tendency for saturation. This result can be related to the diminutive type of forces that govern the mobility of charges under an induced field action. The mentioned behavior is related to the vibration of the ion within the confinement of their potential energy well at a lower frequency, whereas at a higher frequency, it remains insensitive to the applied electric field.^28,29^

**Fig. 9:**
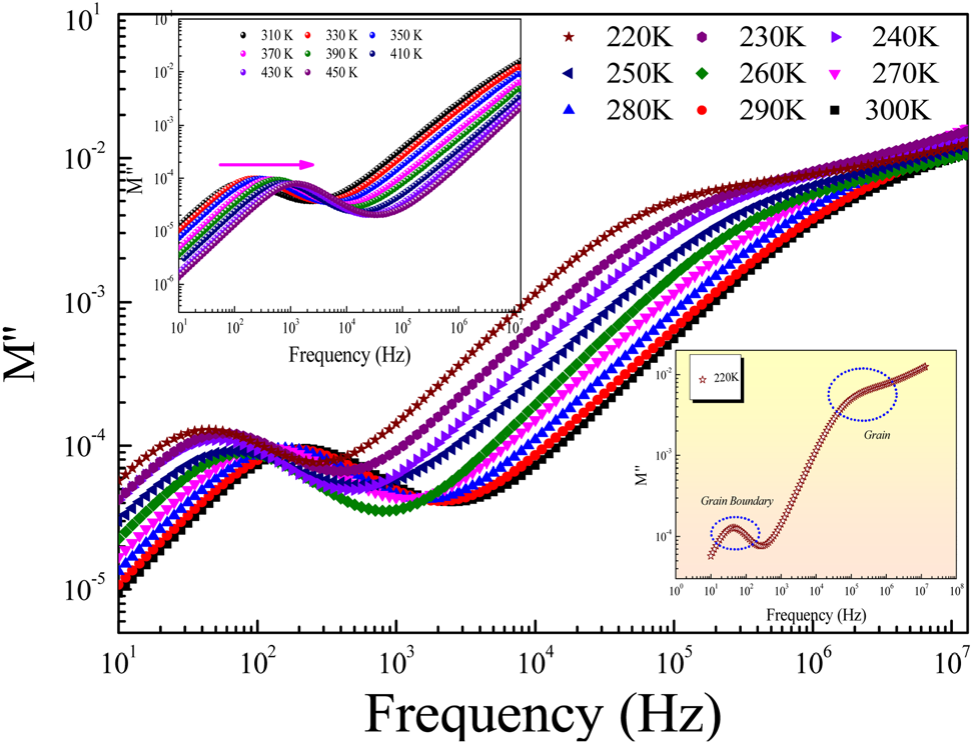
Evolution of the imaginary part *M*′′ of the electric modulus as a function of frequency and over the temperature range from 220 K to 450 K.

**Fig. 10:**
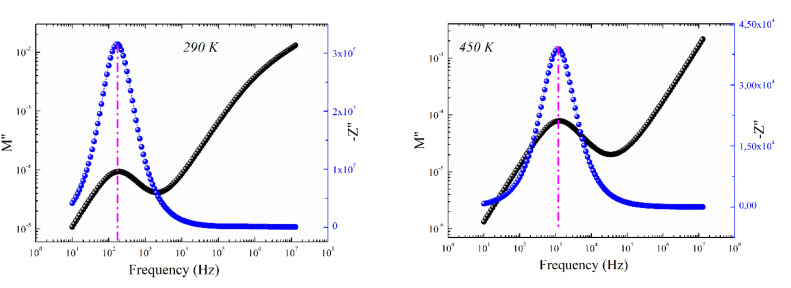
The imaginary part of impedance (*Z*′′) and modulus (*M*′′) *versus* frequencies at selected temperatures (290 and 450 K).

Fig. 9 shows the frequency dependence of the modulus's imaginary part (*M*′′) at various temperatures of the prepared compound. For each temperature value, *M*′′ displays a well-resolved peak at a typical frequency value (*f*_max_). All peaks indicate the existence of conductivity relaxations in the sample. However, the perovskite presents a single peak detected at a low frequency and shifts towards high-frequency values by increasing temperature. This temperature increase is related to the increase in the relaxation rate thanks to the thermal activation of the charge carrier's transport. The temperature dependence of the *M*′′ peak can be related to the studied material's strength and conductivity relaxation process. The appearance of peaks indicates that the charge carriers reveal an evolution from long-range to short-range mobility. For the ideal Debye model, all dipoles must be relaxed with the same relaxation time. However, the existence of non-symmetric *M*′′ peaks confirms the presence of non-Debye-type relaxation.^30^ For some selected temperatures, the combined imaginary modulus (*M*′′) and impedance (*Z*′′) spectra have been designed in log–log scale as shown in Fig. 10. This configuration is adopted to distinguish between the short-range and long-range charge carriers' movement. For the studied GdCaCuO compound, the large mismatch between the *M*′′ maximum and *Z*′′ maximum confirms the localized nature of the conduction process.^31^ In the literature, it is found that the existence of 
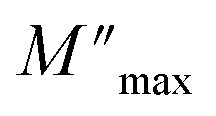
 and 
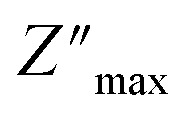
 peaks at the same frequency value indicates that the relaxation process is due to the long-range charge carriers' movement. However, when *f*_max_ (*M*′′) ≠ *f*_max_ (*Z*′′), the charge carrier's movement is related to the short-range mobility.^32,33^ The ion moves over long distances at low frequencies by successfully hopping from one site to the next. However, at the high-frequency range, the presence of a relaxation peak is related to the existence of confined ions to their potential well and can make only the localized motion within the well. For the studied compound and at 290 K and 450 K, the occurrence of *Z*′′ and *M*′′ low-frequency peaks, at the same frequency value, indicates the manifestation of a delocalized relaxation mechanism and long-range mobility.

### 3.4 Conductivity study

**Fig. 11:**
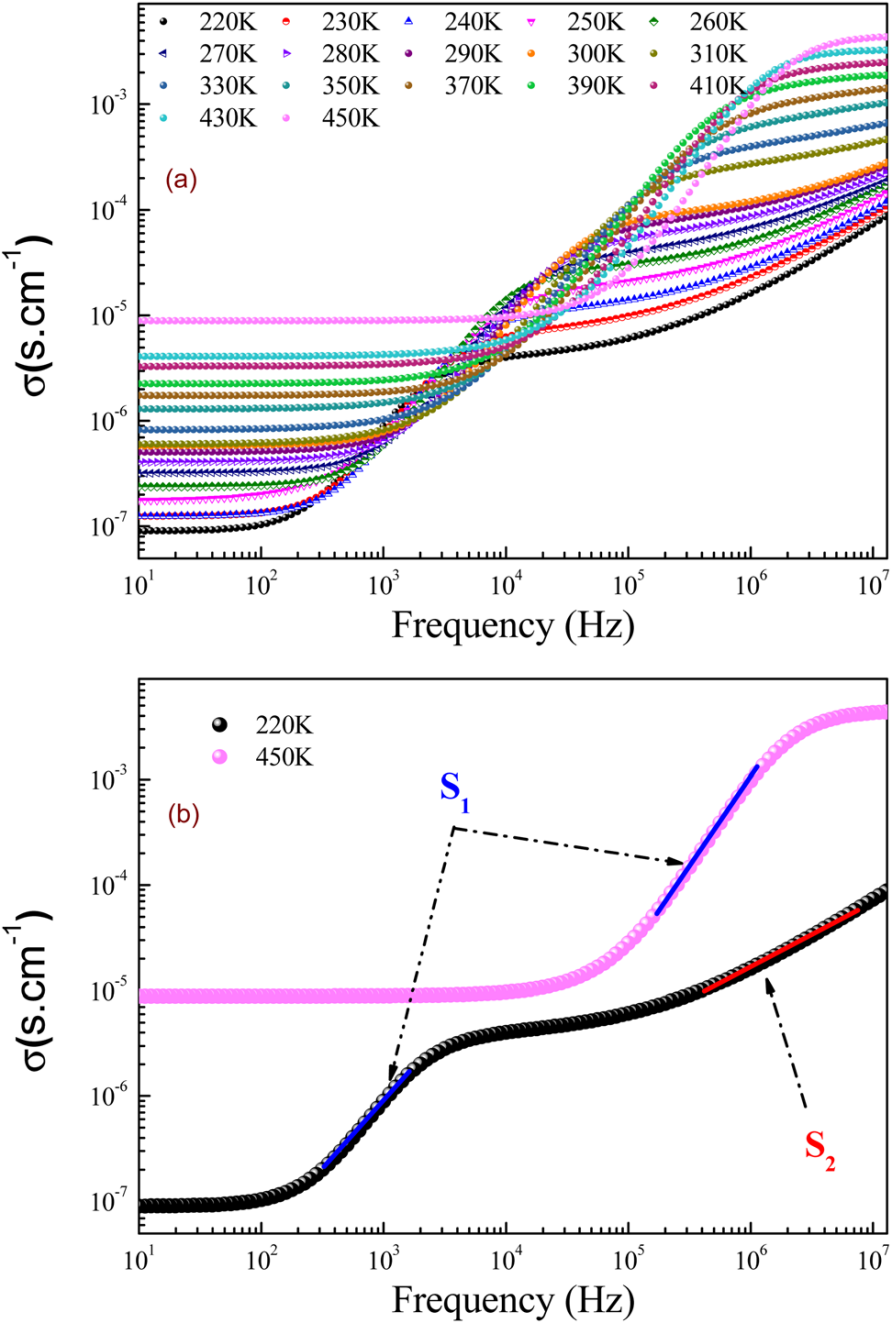
(a and b): Conductivity spectra (*σ*) for the studied GdCaCuO ceramic at different temperatures.

In the preset section, we shall investigate the transport properties of the prepared GdCaCuO perovskite using the conductivity study and some theoretical models. They offer information on the main conduction processes that assure the transport properties of the oxide ceramics. In addition, the electrical conductivity investigation can be employed to identify the electrical properties of oxides. In this work, conductivity data were measured over large frequency and temperature ranges [10 Hz–13 MHz] and [220–450 K], respectively. In the literature, numerous conduction models were employed to describe the dynamic of the charge carriers in the perovskite systems.^15,34^ The total electrical conductivity at a particular temperature contains direct and alternative current components for amorphous, crystalline, and polycrystalline systems. Fig. 11 shows the ac-conductivity (*σ*_ac_) plots *versus* frequency at numerous temperature values. In the present work, each conductivity spectrum can be defined using the double Jonscher law:^14^

7 *σ*(*ω*,*T*) = *σ*_dc_(*T*) + *A*_1_(*T*)*ω*^*s*_1_^ + *A*_2_(*T*)*ω*^*s*_2_^

The first term describes the dc electrical conductivity variation in the studied compound. Hence, the second and the third terms have been introduced to investigate the electrical behavior of the prepared compound in the dynamic region of the spectra (ac-electrical conductivity). The parameters *s*_1_ and *s*_2_ are two frequency exponents, describing the degree of interaction between mobile charges and gives information about the origin of the electrical conduction.

In the limit of the DC range and at low temperatures, the electrical transport properties of the material oxides are largely explained using the variable range hopping conduction process. While, at high temperatures, the small polaron hopping (SPH) conduction process, which is thermally activated, governs the conduction phenomenon.

**Fig. 12:**
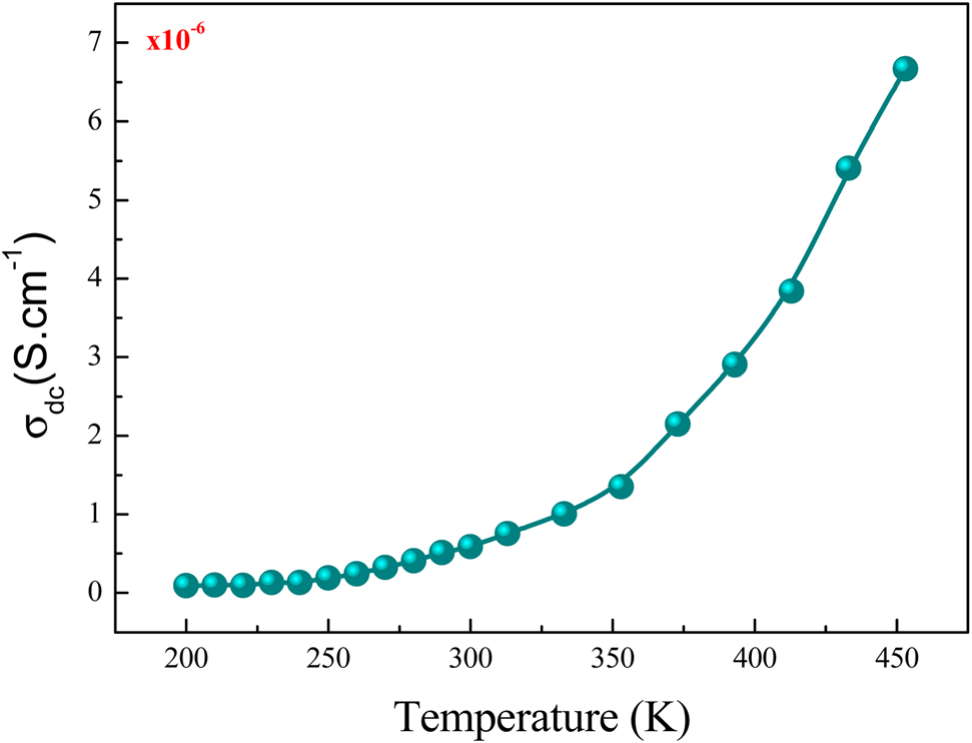
Evolution of the DC conductivity against the temperature for the temperature range of 220 K to 450 K.

**Fig. 13:**
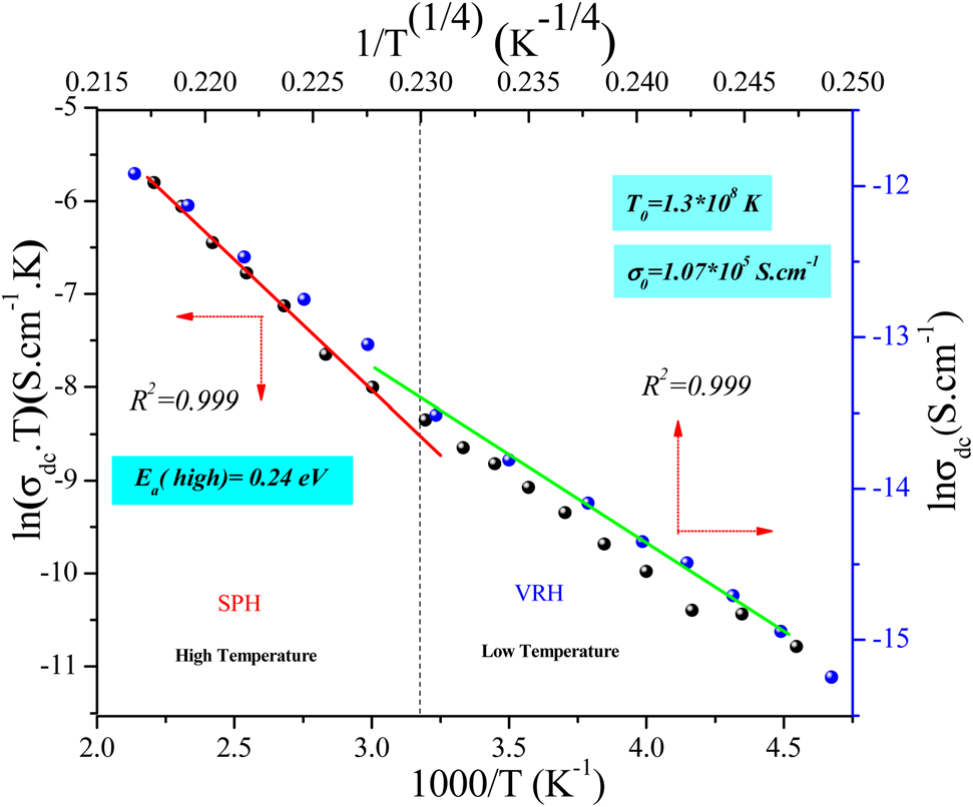
Dependence of ln(*σ*_DC_·*T*) as a function of inverse of temperature (1000/*T*) (bottom and left) and ln(*σ*_DC_) *versus* 1/*T*^(1/4)^ (top and right).

Fig. 12 shows the evolution of the DC-conductivity against the temperature for the studied GdCaCuO sample. Over the whole explored temperature domain, the reported result demonstrates an increase in the electrical conductivity with temperature variation, confirming the semiconductor behavior of the studied compound. The same behavior was observed in various oxide materials like Pr_0.7_Ca_0.3_Mn_0.9_X_0.1_O_3_ manganite.^24^ The activation of the SPH process is related to the formation of small polarons *via* a thermally activated energy called the hopping energy at high temperatures. Fig. 13 (bottom and left) shows the evolution of ln (*σ*_dc_ × *T*) *versus* the inverse of the temperature for the copper GdCaCuO material. At elevated temperatures, the linear curve approves the thermal activation of the SPH mechanism. At this temperature range, the following relation describes the temperature dependence of the DC conductivity:8
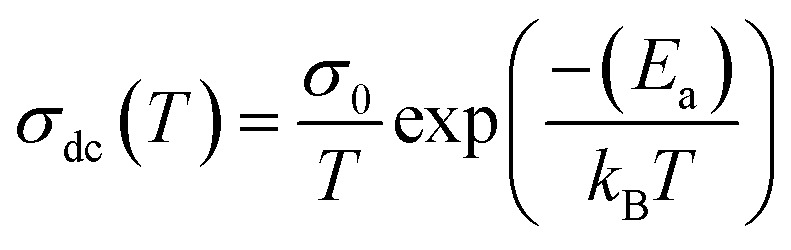


The parameter *σ*_0_ is a pre-exponential factor, which is temperature-independent. *E*_a_ is the activation energy that is needed to move the charge carrier between two conductive sites. The deduced activation energy value at high temperatures is *E*_a_ = 0.24 eV. The activation energy values are close to the measured relaxation energies.^8^ The lower jump activation energy of the oxygen vacancies, which is similar to the reorientation of the dipole, enables dielectric relaxation. The evolution of ln (*σ*_dc_) *versus T*^−1/4^ illustrated in Fig. 13 (top and right) was adopted to show the contribution of the variable range hopping mechanism in the electrical conductivity of the studied compound at low temperatures. For the VRH model, the temperature dependence of the electrical conductivity is defined according to the Mott–VRH law:^16–18^

9 *σ*_dc_ = *σ*_0_e^−(*T*_0_/*T*)1/4^

The linear evolution of ln (*σ*_dc_) *versus T*^−1/4^, at low temperatures (below room temperature), indicates the presence of the hopping of charge carriers between variable sites. The parameters *σ*_0_ and *T*_0_ are found to be 1.07 × 10^7^ S cm^−1^ and 1.3 × 10^8^ K and are in good agreement with other results.^35^ The aforementioned model was adopted to investigate the electrical response of various classes of oxides such as perovskites, spinel ferrites, and thin films. The formation of small polaron charges in copper oxides is due to the hopping of the self-trapped electron on the copper site inside the CuO_2_ planes. The Polaron hopping energy related to *T*_0_ and can be described by the equation:^9^

10 *W* = 0.25*k*_B_*T*_0_^1/4^*T*^3/4^

**Fig. 14:**
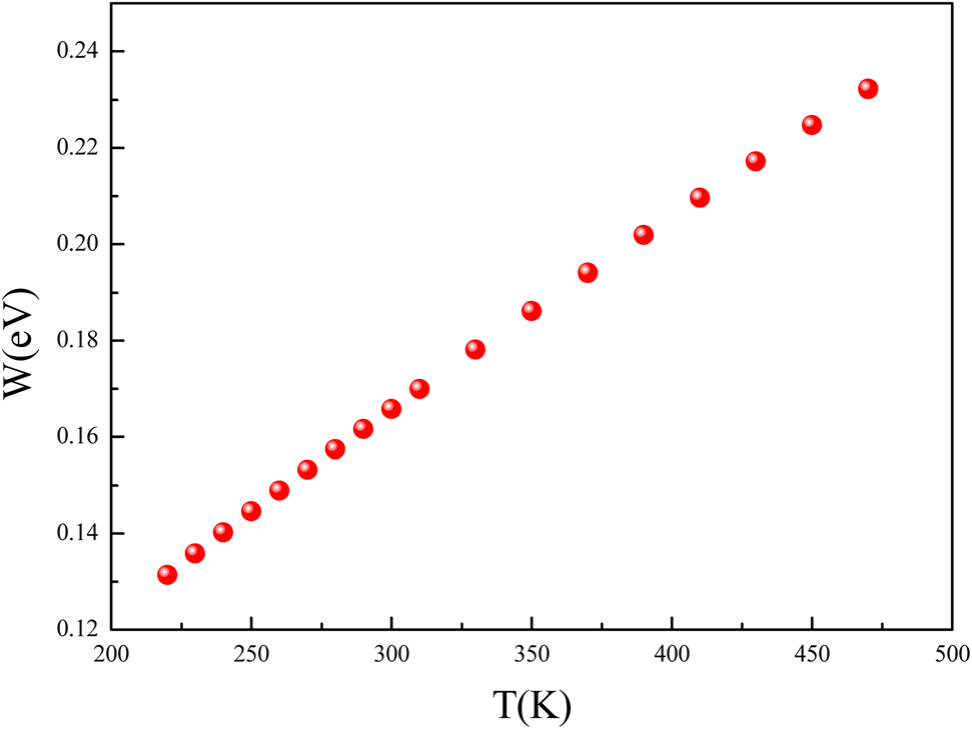
The temperature dependence of the hopping energy *W*.

The temperature dependence of the hopping energy *W* is shown in Fig. 14. The hopping energy *W* increases from 0.13 eV at 220 K to 0.23 eV at 470 K. The carrier's charge is more localized in the low temperature. They are frozen at low temperatures and activated at high temperatures.

For the studied compound, the conductivity spectra have been deduced from the dielectric plots *via* the below relation:^36^

11 *σ*(*ω*,*T*) = *ωε*_0_*ε*′′(*ω*,*T*)

The parameter *ε*_0_ is the electric permittivity of free space charge. Beyond a certain specific frequency, conductivity spectra of the GdCaCuO compound were observed to increase strangely with the frequency increase due to the activation of various conduction mechanisms. To examine the nature of activated conduction processes, in the limit of the ac regime, the frequency dependence of the permittivity was employed. The aforementioned function obeys the Jonscher law, which is defined by the below equations:^9,12,13^12
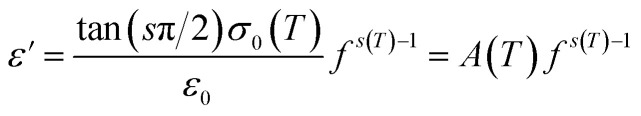


**Fig. 15:**
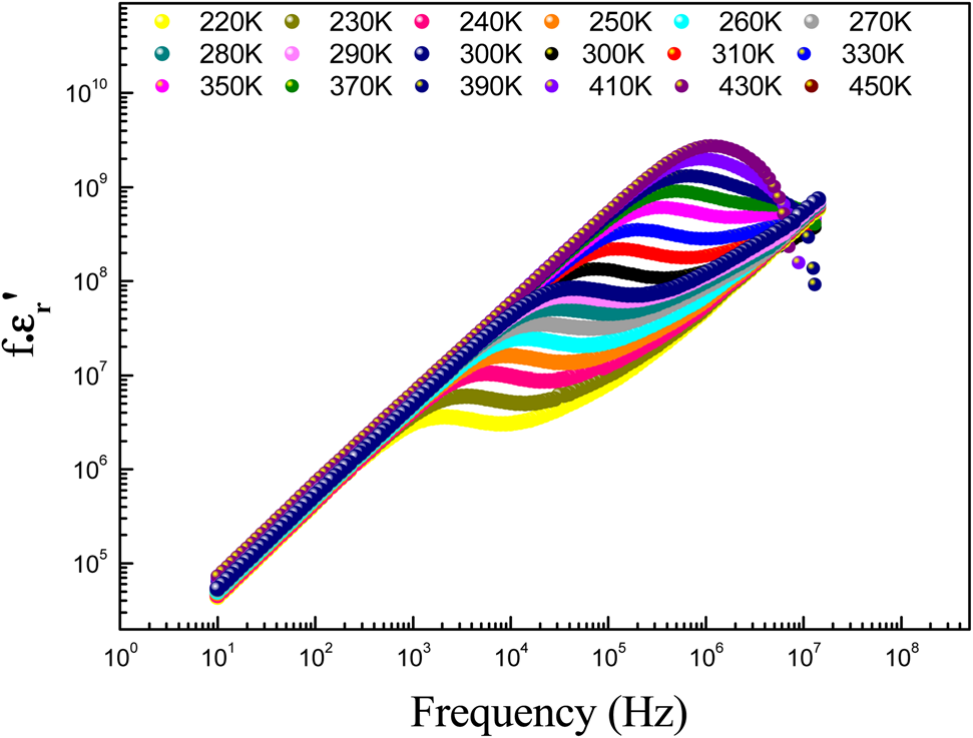
Evolution of (*f*·*ε*′) as a function of frequency at various temperatures.

**Fig. 16:**
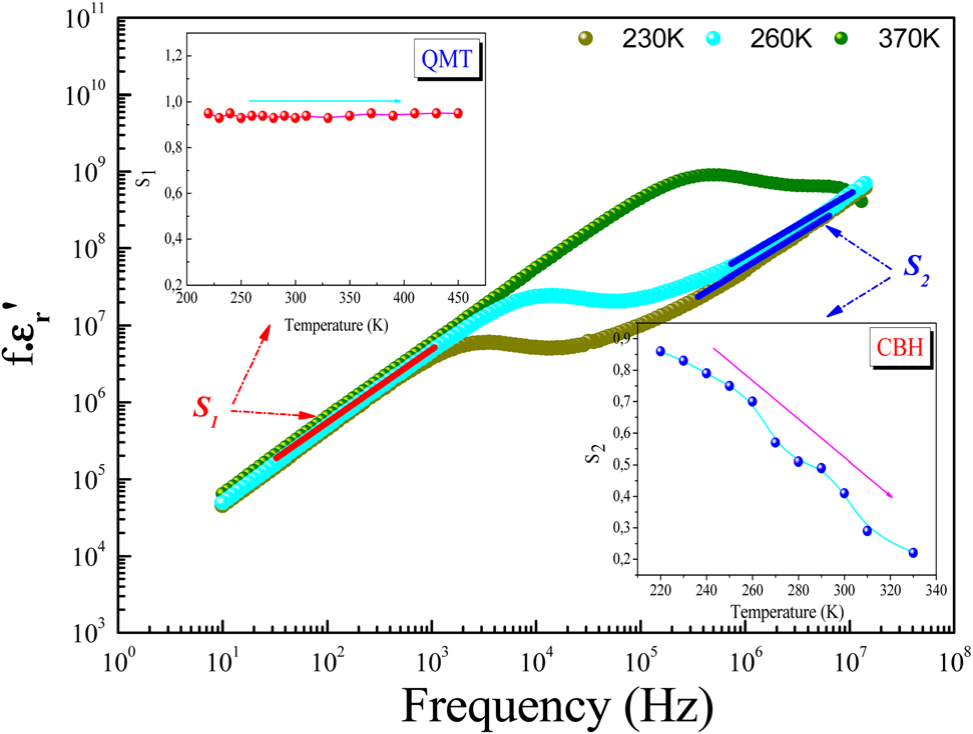
Evolution of the frequency exponents *s*_1_ (a), and *s*_2_ (b) and the deduced conduction mechanisms.

*σ*_0_(*T*) and *s*(*T*) are the temperature-dependent constants, and *ε*_0_ is the electric permittivity of free space charge. Fig. 15 shows the frequency dependence of (*f* × *ε*′) for GdCaCuO ceramic at various temperatures. The informed results show the presence of two power-law variations. Over the explored frequency region, each power-law evolution was associated with the activation of one or more conduction processes. Thus, the linear slopes of (*f* × *ε*′) spectra are used to estimate temperature dependence for frequency exponents *s*_1_ and *s*_2_, which are mainly employed to characterize the conduction mechanisms in the AC region of spectra.^37–41^ From the linear fits, the obtained frequency exponent *s*_1_ is exposed in the inset of Fig. 16. This value indicates that the Quantum Mechanical Tunneling (QMT) conduction process governs the electrical conductivity.^37–41^ The temperature dependence of the second frequency exponent *s*_2_ (inset Fig. 16) confirms the contribution of the correlated barrier hopping mechanism (CBH) to the transport proprieties of the studied compound. For the CBH model, the frequency exponent has decreased against increasing the temperature. The conduction mechanism in the CBH model involves the transfer of charge through a polaron hopping process transversely to the Coulomb barrier that separates two defect centers. On the other hand, the quantum mechanical tunneling (QMT) model assumes the tunneling of charge carriers between two localized states.^42^

## 4 Conclusion

The correlation of polaron conduction and colossal permittivity of GdCaCuO is established by a modified equivalent circuit based on the internal barrier layer (IBL) model, which is well-confirmed by the experimental data. The impedance study confirms the presence of a non-Debye electrical relaxation process in the compound. In addition, it proves the important contribution of both grain and grain boundary zones. The combined study of the electric modulus and the impedance results indicate that the charge carrier's movement in the GdCaCuO compound is related to short-range mobility. At high frequencies, the presence of relaxation peaks *M*′′ and *Z*′′ is related to the existence of confined ions to their potential well and can make only the localized motion within the well. The studied copper compound displays a semi-conductor character in the whole studied temperature region. The electrical transport was attributed to the activation of the small polaron hopping at high temperatures and the variable range hopping processes at low temperatures. As temperature increases from 220 K to 470 K, their hopping energy increases from 0.13 to 0.23 eV. The conductivity spectra have been analyzed by the double Jonscher power law. From the frequency exponent's variations, the ac electrical properties of the studied system are described by the activation of correlated barrier hopping and the quantum mechanical tunneling conduction processes. The quantum mechanical tunneling (QMT) model assumes the tunneling of charge carriers between two localized states near the Fermi level, the correlated barrier hopping mechanism assumes that the charge carrier hops from site to site over the potential barrier.

## Data availability

The data that support the findings of this study are available from the corresponding author upon reasonable request.

## Conflicts of interest

The authors declare that they have no known competing financial interests or personal relationships that could have appeared to influence the work reported in this paper.

## Supplementary Material
